# Report of a Case of Carcinoma of the Buccal Mucous Membrane with Microscopic Exhibit

**Published:** 1898-01

**Authors:** Geo. J. Dennis

**Affiliations:** Chicago, Ill.


ARTICLE V.

### REPORT OF A CASE OF CARCINOMA OF THE BUCCAL MUCOUS MEMBRANE WITH MICROSCOPIC EXHIBIT.

BY GEO. J. DENNIS, M. D., D. D. S., CHICAGO, ILL.

Read before the Odontographic Society of Chicago, Oct., 1897.

In presenting a report of a cancer of the cheek, it is not with the idea of presenting anything new, or unusual, but it is done because of the interest of the case to the writer, and because he believes that a description of the case and the operation for the removal of the tumor will be of interest to the members of this society; sufficient at least to serve as an excuse for the presentation of a case, and an operation at which he was only a spectator and not an active participant.

The operation for the removal of the tumor was performed at Wesley Hospital by Dr. Wm. E, Schroeder, of this city, assisted by Dr. S. C. Plummer, and it is through Dr. Schroeder's kindness that the patient and the microscopic exhibit are here this evening.

The report is as follows: Mr. R., aged fifty-four. Family habits, negative. Personal history: Habits: Uses tobacco moderately, smokes occasionally, chews on the left side and drinks alcoholics very moderately.

Previous diseases. The usual diseases of childhood, scarlet fever, measles, etc., and one attack of pneumonia. Otherwise has always been well.

Present illness. Began three and one-half months before the operation in the form of a small swelling in the middle of the right buccal mucous membrane. It grew rapidly with little pain and soon ulcerated. The odor from the mouth increased as the growth increased. During the last few weeks the patient noticed a swelling under the lower jaw.

Examination. In the right side of the mouth a tumor about three-quarters of an inch below the opening of Steno's duct of the size of a silver dollar, extending downward and involving the mucous membrane of the inferior maxilla. The surface was ulcerated, the edges indurated and irregular. Under the right inferior maxilla several glands, one of which was the submaxillary lymph gland, were palpated. The cervical glands were not enlarged. Submental glands were also negative. Of the teeth, the second bicuspid, first and second molars were missing, the others in good condition.

Physical examination, negative; the patient somewhat cachectic. Urine negative. A specimen was removed from the border of the tumor hardened in alcohol, formalin and water for six days, mounted, sections cut and stained. The microscopic examination revealed an alveolar carcinoma.

Preparation for operation. Consisted of a mouth wash for one week of a saturated solution of boric acid; on the day before the operation the patient was scrubbed, washed and shaved about neck and face; a wet boric acid dressing applied and only liquid food permitted.

The operation. Anæsthetic, chloroform during the early stages followed by ether, with a return to chloroform at the close.

To avoid paralysis of the facial nerve, the lower lip was split in the median line between two Billroth forceps to the lower edge of the chin by means of scissors and the incision carried on a line parallel with the lower border of the jaw to a point about three quarters of an inch from the mastoid process. Thc hæmorrhage was arrested by tying the facial artery, veins aud branches. The side of the cheek was then lifted, and the tumor and neighboring tissue three and one half by two inches removed, the incision being carried about three-fourths of an inch outside of the mass and as high as Steno's duct. The third molar, first bicuspid and cuspid were extracted, and the mucous membrane of the outer portion of the jaw and the alveolar process were removed. The hæmorrhage from the jaw was stopped by means of the Paquelin cautery. The submaxillary lymph and salivary glands as well as all the other submaxillary glands were then removed because adherent. The submental glands were searched for, but none were found. These are not always present (Henle).

A measure of the denuded surface was then taken with gutta-percha tissue, and a flap was dissected from the neck and infra-clavicular region leaving a pedicle sufficient for the nutrition of the detached skin. After this the flap was carried up and stitched into the denuded area of the cheek. The cheek was then brought forward to place and the first incision through the lip and along the jaw was closed by interrupted sutures, except that portion through which the flap from the chest was carried. Retention sutures were applied to the denuded area over the chest to diminish the wound as much as possible and the remaining uncovered surface received a covering of iodoform gauze. The wound was now dressed aseptically with the head drawn toward the right shoulder to lessen the tension on the flap. The dressings were changed frequently because of the passage of secretions from the mouth between the flap and jaw.

Twelve days later the flap from the chest was incised from its pedicle near the opening into the mouth and returned to its place on the chest; the separated portion was carried inside of the mouth and sutured to the mucous membrane at the side of the tongue, and the original opening along the jaw which had been only partially closed was now completely closed. The remaining uncovered surface of the chest was filled in with Thierch skin grafts.

It is due to Dr. Schroeder to state that another operation for the removal of the cervical glands will take place in order that metastatic growths in other parts of the body may be prevented.—*Dental Review.*

